# The dissection of *R* genes and locus *Pc5.1* in *Phytophthora capsici* infection provides a novel view of disease resistance in peppers

**DOI:** 10.1186/s12864-021-07705-z

**Published:** 2021-05-21

**Authors:** Jin-Song Du, Lin-Feng Hang, Qian Hao, Hai-Tao Yang, Siyad Ali, Radwa Salah Ezaat Badawy, Xiao-Yu Xu, Hua-Qiang Tan, Li-Hong Su, Huan-Xiu Li, Kai-Xi Zou, Yu Li, Bo Sun, Li-Jin Lin, Yun-Song Lai

**Affiliations:** College of Horticulture, Sichuan Agricultural University, Chengdu, 611130 China

**Keywords:** Root rot, Disease resistance, *R* gene, NBS-ARC domain, RNA-seq

## Abstract

**Background:**

*Phytophthora capsici* root rot (PRR) is a disastrous disease in peppers (*Capsicum spp*.) caused by soilborne oomycete with typical symptoms of necrosis and constriction at the basal stem and consequent plant wilting. Most studies on the QTL mapping of *P. capsici* resistance suggested a consensus broad-spectrum QTL on chromosome 5 named *Pc.5.1* regardless of *P. capsici* isolates and resistant resources. In addition, all these reports proposed NBS-ARC domain genes as candidate genes controlling resistance.

**Results:**

We screened out 10 PRR-resistant resources from 160 Capsicum germplasm and inspected the response of locus *Pc.5.1* and NBS-ARC genes during *P. capsici* infection by comparing the root transcriptomes of resistant pepper 305R and susceptible pepper 372S. To dissect the structure of *Pc.5.1*, we anchored genetic markers onto pepper genomic sequence and made an extended *Pc5.1* (*Ext-Pc5.1*) located at 8.35Mb38.13Mb on chromosome 5 which covered all *Pc5.1* reported in publications. A total of 571 NBS-ARC genes were mined from the genome of pepper CM334 and 34 genes were significantly affected by *P. capsici* infection in either 305R or 372S. Only 5 inducible NBS-ARC genes had LRR domains and none of them was positioned at Ext*-Pc5.1*. *Ext-Pc5.1* did show strong response to *P. capsici* infection and there were a total of 44 differentially expressed genes (DEGs), but no candidate genes proposed by previous publications was included. *Snakin-1* (*SN1*), a well-known antimicrobial peptide gene located at *Pc5.1*, was significantly decreased in 372S but not in 305R. Moreover, there was an impressive upregulation of sugar pathway genes in 305R, which was confirmed by metabolite analysis of roots. The biological processes of histone methylation, histone phosphorylation, DNA methylation, and nucleosome assembly were strongly activated in 305R but not in 372S, indicating an epigenetic-related defense mechanism.

**Conclusions:**

Those NBS-ARC genes that were suggested to contribute to *Pc5.1* in previous publications did not show any significant response in *P. capsici* infection and there were no significant differences of these genes in transcription levels between 305R and 372S. Other pathogen defense-related genes like *SN1* might account for *Pc5.1*. Our study also proposed the important role of sugar and epigenetic regulation in the defense against *P. capsici*.

**Supplementary Information:**

The online version contains supplementary material available at 10.1186/s12864-021-07705-z.

## Background

Oomycete *Phytophthora capsici* is a soilborn pathogen fungus that causes fruit rot, stem blight, foliar blight, and particularly root rot in peppers depending on the disease occurrence position [1]. *P. capsici* root rot (PRR) is a devastating pepper disease with typical symptoms of necrosis and constriction at the basal stem and consequent plant wilting. *P. capsici* basically spread via soil and splashing water in the form of microzoospores, and the disease PRR can break out very quickly in summer 12days after rainfall due to field ponding. In protected cultivation, root rot occurs frequently 12months after transplantation, especially in the case of continuous cropping. Resistance breeding is the first choice to prevent disease damage. A resistance genetic source PI201234 was first found in pepper [2]. The best known source is Criollo de Morelos-334 (CM334), which is also the sequencing material due to its perfect resistance [3]. It is still important to explore more genetic resources of *P. capsici* resistance.

Disease PRR was first reported in 1918 in the US, which was later found to be caused by *P. capsica*, a new fungus species [4]. Hence, phytopathologists and microbiologists have made great efforts to understand its pathogenic features [5]. *P. capsici* has even become a model pathogen in the study of plant-microbe interactions due to its wide range of hosts, including potato, tomato, cucurbits, beans, *Arabidopsis* and tobacco [6,8]. On the attack side, *Phytophthora* pathogens secrete and dispatch effectors such as RxLR into host cells, which paralyse the plant host immune system, including basal immune system named pattern-triggered immunity (PTI) [9], endoplasmic reticulum (ER) stress-mediated plant immunity [10], and the EDS-PAD4 immune signaling pathway [11]. In addition, pathogenic effectors can also disturb histone acetylation [12] and ethylene biosynthesis [13].

On the other side of host defense, plants develop PTI to detect nonspecific pathogen/microbe-associated molecular patterns (P/MAMPs), and effector-triggered immunity (ETI) which is resistance specific and accompanied by a hypersensitive response (HR) [14]. In the ETI system, NBS (nucleotide binding site)-ARC (apoptosis, R proteins, CED-4)-LRR (leucine rich repeat) proteins recognize pathogenic effectors and trigger downstream defense processes, including a rapid and strong oxidative burst, pathogenesis-related (PR) gene expression, and accumulation of antimicrobial compounds. NBS-ARC-LRR protein genes constitute the predominant majority of disease resistance genes (*R* genes). Several doses of *R* genes have been amplified in peppers by degenerate primers [15, 16]. However, most *R* genes are still unknown because higher plants typically have hundreds of *R* genes. As demonstrated in potato [17] and many other higher plants, pepper *R* gene proteins should also optionally have conserved domains of toll/interleukin-1 receptor (TIR), coiled-coil (CC), and resistance to powdery mildew 8 (RPW8) in addition to NBS, ARC and LRR. Among these above domains, the NBS-ARC domain is the most conserved and is widely used to identify *R* genes.

QTL mapping of pepper resistance to *P. capsici* was first reported in 1996 [18]. In this study, 13 QTLs were identified using the F2 mapping population of Perennial and YOLO Wonder, and one QTL linked to molecular marker TG483 on chromosome 5 had a major effect on resistance, which explained 4155% of the total variance. Since then, many studies have confirmed these QTLs on chromosome 5 using different genetic resources (mostly CM334), mapping populations, and mapping strategies [19,23]. Based on the above studies, Mallard et al. (2013) constructed three consensus QTLs on chromosome 5 by using anchor markers and meta-analysis [24]. *Meta-Pc5.1* and *Meta-Pc5.3* were positioned close to teach other on the short arm of the chromosome and *Meta-Pc5.2* was on the long arm. Recent QTL mapping work again confirmed the major QTL on the short arm of chromosome 5 [25,27]. Now, it is very clear that the major QTL *Pc5.1* is a broad-spectrum QTL that controls resistance to all *P. capsici*. All the reports proposed *R* genes at *Pc5.1* as candidate genes. However, the detailed genetic mechanism remains unknown, and the function of these *R* genes needs to be characterized. The pepper genome sequences of CM334 and Zunla were independently released in 2014 [28, 29], which enabled a thorough dissection of QTL structure.

In this study, we identified NBS-ARC candidate genes by mining the genomic sequence and profiled the responses of these genes in *P. capsici* infection. We also constructed an extended *Pc5.1* (*Ext-Pc5.1*) to cover all reported QTLs from different QTL mapping works and profiled the responses of the genes on this locus in *P. capsici* infection. The comparison of root metabolites and root transcriptome between resistant and sensitive peppers in *P. capsici* infection renewed our understanding about the roles of *R* genes and QTL *Pc5.1*and provide new insights in *P. capsica*-resistance.

## Results

### Resistance assessment of *Capsicum* germplasm

**Fig. 1:**
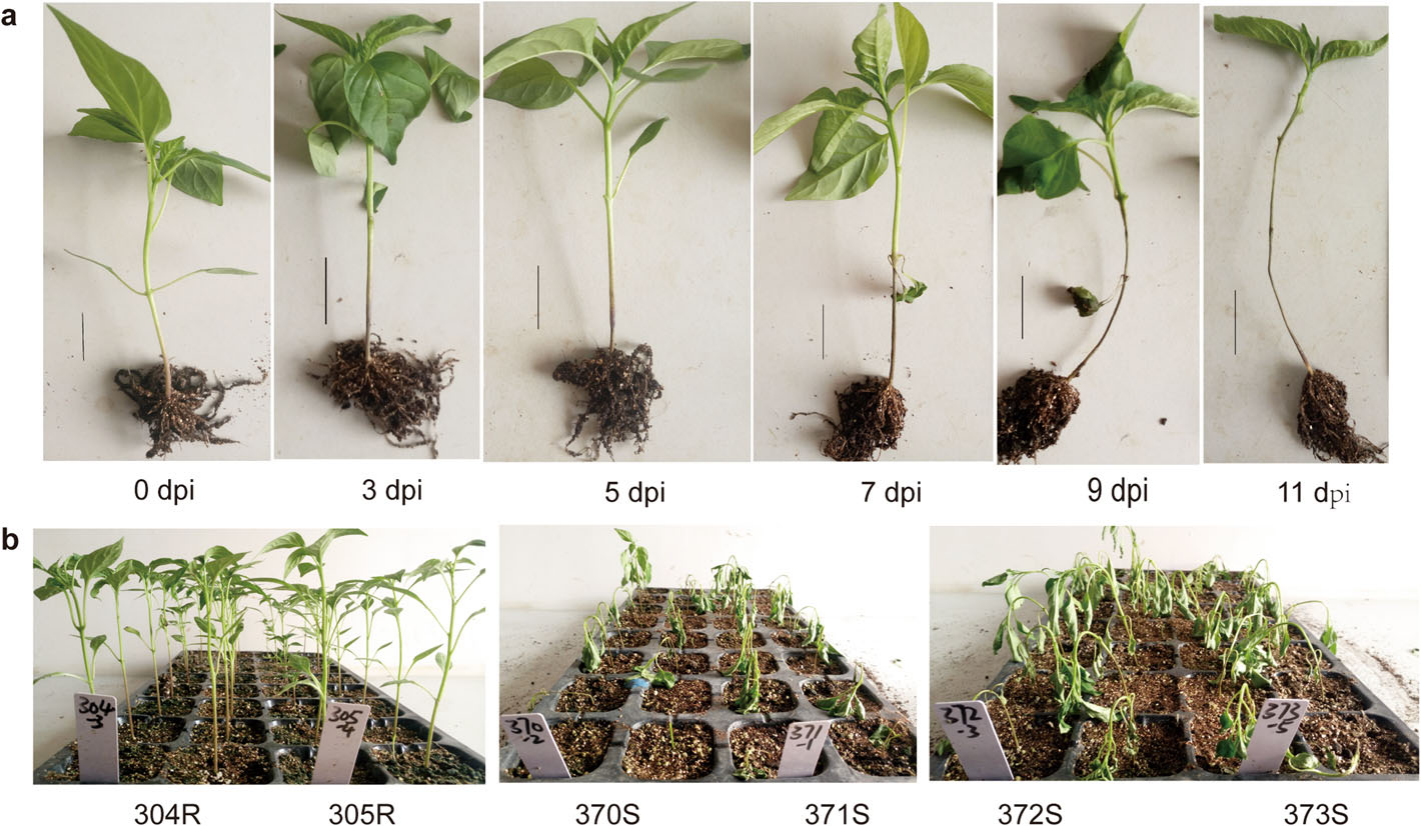
Symptom of *P. capsici* root rot (PRR). **a** Dynamic symptom after root inoculation. **b** Comparison of *P. capsici* resistance between resistant and susceptible pepper materials at 7days post inoculation (dpi)

Pepper seedlings with 6 leaves were inoculated with *P. capsici* by injecting zoospores into the soil around the basal stem (Fig.1). A total of 160 germplasm materials were subjected to the resistance assessment. As a result, we identified 10 materials of high resistance (HR), 7 materials of resistance (R), 31 materials of moderate resistance (MR), and 112 materials of nonresistance (NR) (Additionalfile1: Table S1). The HR pepper germplasm showed comparable resistance to CM334. The ten HR germplasms included 2 bell peppers, 5 cayenne peppers, 1 cluster pepper, 1 linear pepper (var. annuum L. dactylus M), and 1 upward pepper (var. conoide (Mill.) Isish). We selected four accession (304R, linear pepper; 305R, upward pepper; 370S, cone pepper; 372S, cayenne pepper) to be used in the following experiments that represented different pepper types.

### Primary metabolites in infected roots

**Fig. 2:**
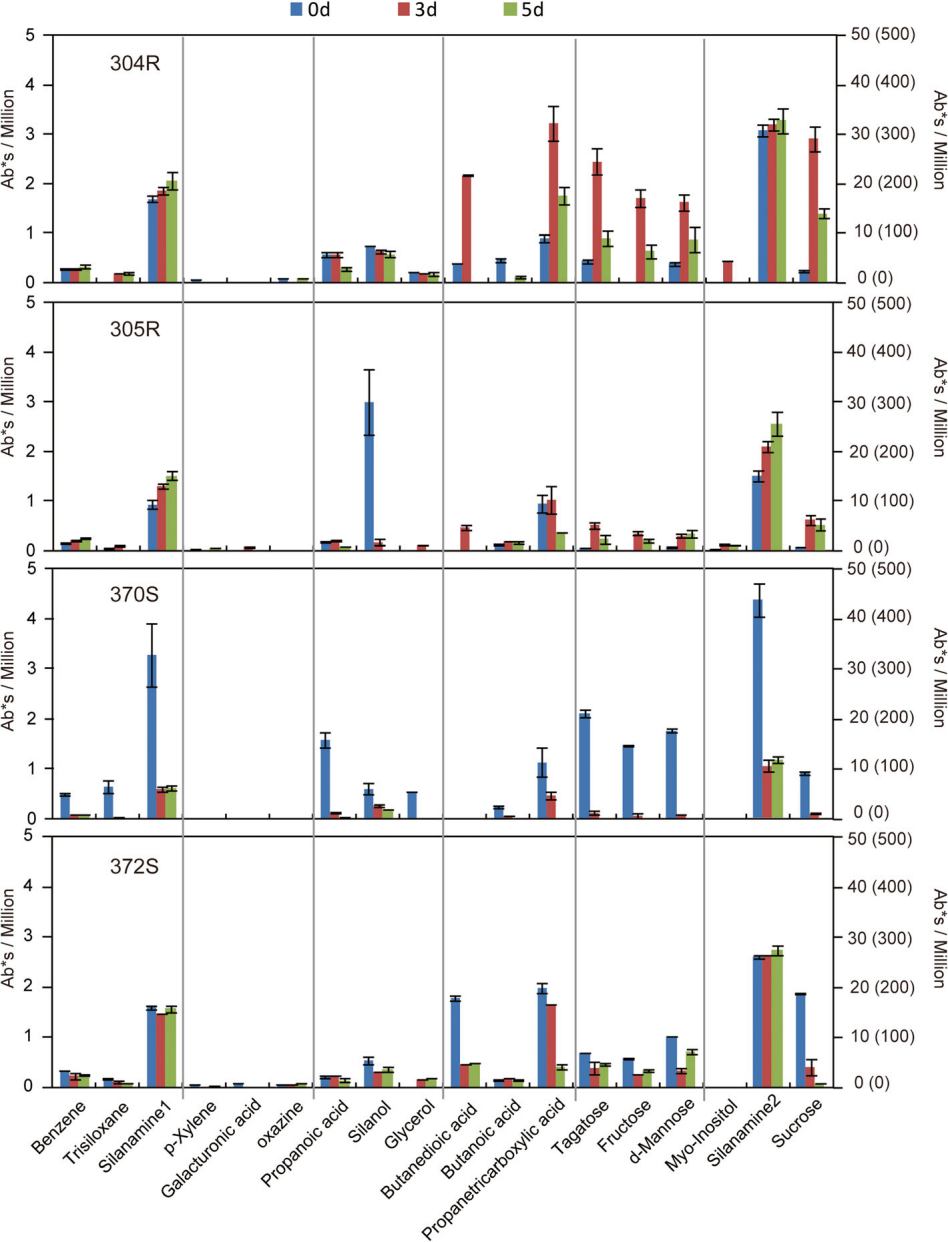
Dynamic profiles of partial metabolites detected in roots were compared between resistant and susceptible peppers. The compound of each metabolite is simply measured by peak area. Sucrose matches the value of the right y-axis outside the parentheses. Silanamine2 matches the value of the right y-axis inside the parentheses. The remaining compounds match the left y-axis. The column bar indicates SE

Ethanol extract from inoculated roots was subjected to GC-MS, which revealed dynamic changes in primary metabolites responding to *P. capsici* infection (Fig.2). All inner standards were salinized, which indicates total and successful derivatization. Resistant accessions 304R and 305R show different alteration profiles to 370S and 372S in respect of sugar contents. There was a sharp increase of sucrose at 3days post inoculation (3 dpi) in resistant peppers but decrease in susceptible peppers. Similarly, tagatose, fructose and mannose were strongly increased in 304R and 305R but decrease in susceptible peppers particularly 370S. In addition, propanetricarboxylic acid and butanedioic acid were reduced quickly after *P. capsici* inoculation in 370S and 372S but not in resistant materials. No additional consensus differences between resistant peppers and susceptible peppers were observed for the remaining compounds. The robust response of sugar contents may enhance the resistance against *P. capsici*.

### Transcriptome of infected roots

**Fig. 3:**
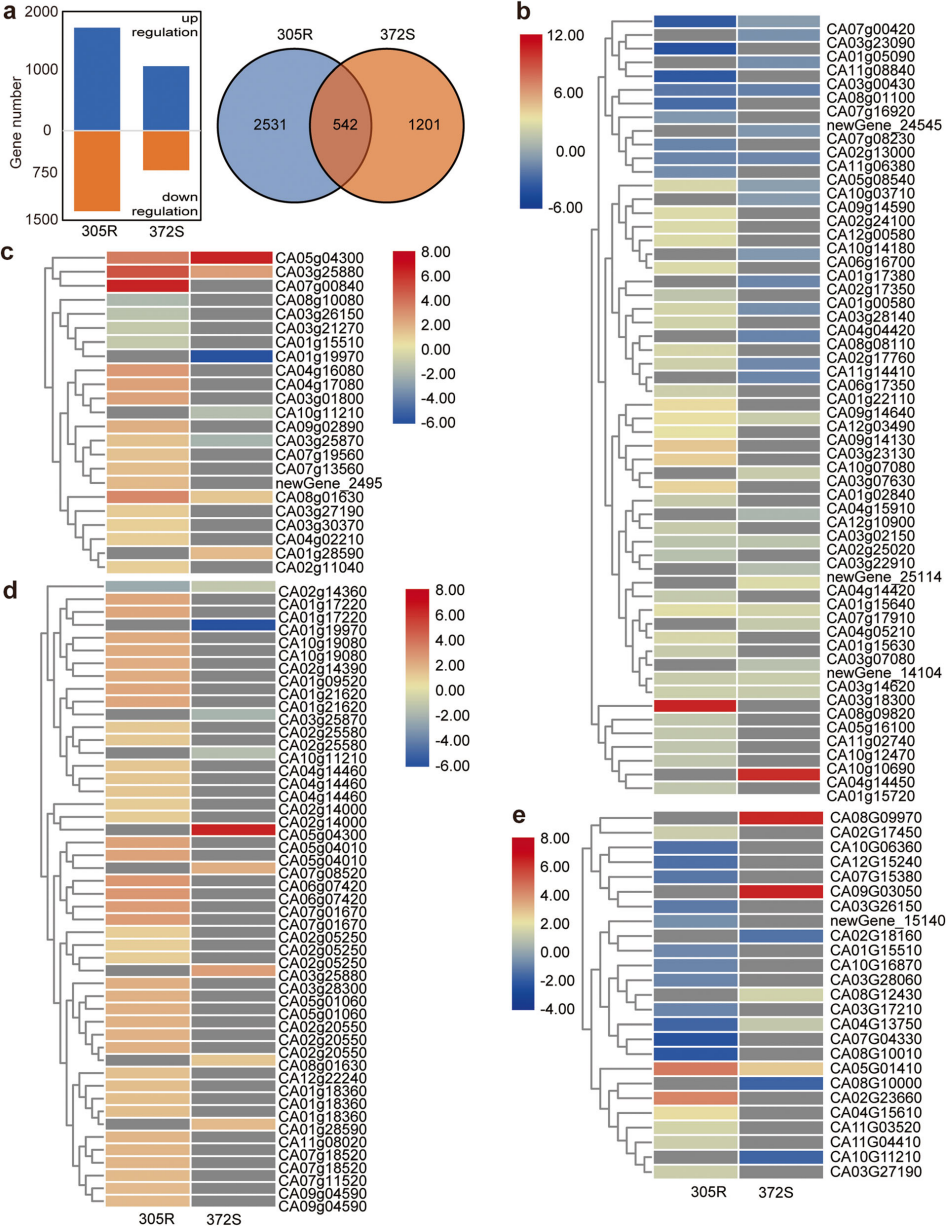
Differential response of gene expression to *P. capsici* infection at 3days post inoculation (dpi). **a** Change trend (left) and Venn diagram (right) of DEGs in pepper 305R and 372S. Genes involved in starch and sucrose metabolism (ko00500, **b**) as well as biological processes related to the ER stress response (GO0034976, **c**), epigenetic regulation (**d**), and fungal response (**e**) in GO analysis show different responses to infection. The color of the heatmap indicates the value of log_2_ (FPKM-3 dpi/FPKM-0 dpi)

We performed RNA-seq using roots of 305R and 372S to profile the dynamic response of the major QTL and NBS-LRR genes that may contribute to resistance against *P. capsici* (Additionalfile2: Table S2). The transcriptome at 3 dpi was compared with that at 0 dpi to identify differentially expressed genes (DEGs) caused by *P. capsici*. As a result, a total of 3073 and 1743 DEGs were identified in 305R and 372S, respectively (Fig.3a; Additionalfile3: Table S3; Additionalfile4: Table S4). For both 372S and 305R, there were more upregulated DEGs than downregulated DEGs. There were many more DEGs in 305R than in 372S, indicating a strong defense response in 305R. This finding is interesting when considering that a visible symptom was noted for 372S, but no change in appearance was noted for 305R.

In KEGG enrichment analysis, the largest differences were noted in the pathways of valine, leucine and isoleucine degradation (ko00280, downregulated) and starch and sucrose metabolism (ko00500, upregulated) in 305R as well as carotenoid biosynthesis (ko00906, upregulated) and plant hormone signal transduction (04075) in 372S (Table1; Additionalfile8: Figure S1). Pathogen infection repressed the expression of ethylene signal transduction genes in 372S and disturbed other phytohormone signal pathways, including auxin, cytokinine, gibberellin, abscisic acid, brassinosteroid, jasmonic acid, and salicylic acid. Significant enrichments of both phenylpropanoid biosynthesis and glutathione metabolism were found in 305R and 372S.

**Table 1:** Pathways showing significant enrichment in KEGG analysis

Pathway	KO ID	EF^a^	Q-value	Number of DEGs	
				305R	372S
Root transcriptome of 305R					
Phenylpropanoid biosynthesis	ko00940	1.815	0.034	35	25
Glutathione metabolism	ko00480	2.019	0.105	22	19
Valine, leucine and isoleucine degradation	ko00280	2.225	0.178	16	
Starch and sucrose metabolism	ko00500	1.567	0.214	42	24
Root transcriptome of 372S					
Glutathione metabolism	ko00480	3.209	0.000	22	19
Carotenoid biosynthesis	ko00906	4.406	0.001		12
Phenylpropanoid biosynthesis	ko00940	2.386	0.004	35	25
Plant hormone signal transduction	ko04075	2.045	0.009		31

^a^Enrich Factor

In the GO enrichment analysis, only 3 significant enrichments were shared by 305R and 372S, indicating very different responses of the transcriptome to *P. capsici* (Table2; Additionalfile9: Figure S2). Notably, 20 DEGs in 305R were enriched under the GO term response to endoplasmic reticulum (ER) stress, whereas that number was 7 in 372S, indicating a differential response in ER stress-mediated plant immunity. As a successful defense, 305R also shows an impressive response inside nuclear processes including nucleosome assembly and DNA replication initiation, epigenetic processes including, chromatin silencing by small RNA, methylation-dependent chromatin silencing, histone methylation and phosphorylation, and DNA methylation. A total of 117 genes were assigned with epigenetic-related biological processes among which 42 genes were significantly affected by *P. capsici* in 305R while that number was 4 in 372S (Additionalfile5: Table S5). We found many interesting DEGs responding to *P. capsici* in 305R, e.g., Histone, ATP-dependent DNA helicase, Chromatin structure-remodeling complex protein, NBS-LRR and Pentatricopeptide repeat-containing protein that may generate phasiRNAs in dicots [30].

**Table 2:** Significant enrichment of biological processes in GO enrichment analysis

GO_ID	GO_Term	Total gene number	Expected DEG number	DEG number	
				305R	372S
Root transcriptome of 305R					
GO:0008283	Cell proliferation	78	9.42	29	2
GO:0042542	Response to hydrogen peroxide	82	9.91	19	6
GO:0051567	Histone H3-K9 methylation	48	5.8	16	1
GO:0019684	Photosynthesis, light reaction	269	32.5	9	2
GO:0006334	Nucleosome assembly	43	5.2	20	2
GO:0016572	Histone phosphorylation	17	2.05	8	0
GO:0006270	DNA replication initiation	29	3.5	13	1
GO:0043086	Negative regulation of catalytic activity	104	12.57	20	22
GO:0006306	DNA methylation	60	7.25	17	2
GO:0034976	Response to endoplasmic reticulum stress	81	9.79	20	7
GO:0006346	Methylation-dependent chromatin silencing	23	2.78	6	1
GO:0009644	Response to high light intensity	53	6.4	18	6
GO:0022900	Electron transport chain	383	46.27	7	1
GO:0042777	Plasma membrane ATP synthesis coupled proton transport	44	5.32	0	0
GO:0009664	Plant-type cell wall organization	96	11.6	18	6
GO:0031048	Chromatin silencing by small RNA	16	1.93	5	0
GO:0009408	Response to heat	83	10.03	24	0
GO:0045893	Positive regulation of transcription, DNA-templated	107	12.93	15	5
GO:0043687	Posttranslational protein modification	28	3.38	2	1
Root transcriptome of 372S					
GO:0043086	Negative regulation of catalytic activity	104	6.36	20	22
GO:0016099	Monoterpenoid biosynthetic process	11	0.67	0	1
GO:0042777	Plasma membrane ATP synthesis coupled proton transport	44	2.69	0	0
GO:0009411	Response to UV	63	3.85	10	4
GO:0055114	Oxidation-reduction process	1865	114.1	206	146
GO:0006355	Regulation of transcription, DNA-templated	647	39.58	95	55
GO:0010035	Response to inorganic substance	338	20.68	59	41
GO:0042542	Response to hydrogen peroxide	82	5.02	19	12
GO:0009825	Multidimensional cell growth	40	2.45	10	2
GO:0009908	Flower development	201	12.3	27	7
GO:0043687	Posttranslational protein modification	28	1.71	2	1

All the biological processes had a *ks* value <0.001

**Fig. 4:**
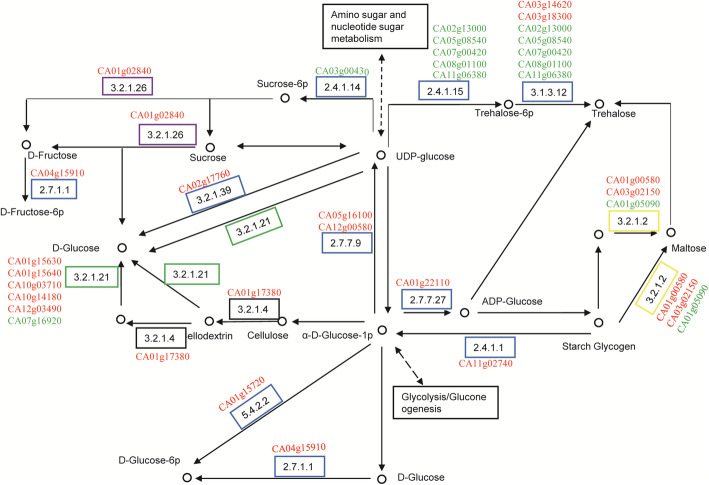
The dynamic change of sugar pathway genes after *P. capsici* infection in 305R. The pathway map was made based on map00500 (Starch and sucrose metabolism) from KEGG PATHWAY Database. Upregulated genes were labeled in red color while downregulated genes were labeled in green color

Based on the results above of the enrichment analysis, we further compared the expression of DEGs under several interesting KEGG pathways or GO terms (Fig.3b-e). DEGs involved in endoplasmic reticulum stress and epigenetic modification were notably upregulated in 305R. Interestingly, under the GO term fungus response, 12 DEGs out of 19 DEGs were downregulated in 305R, whereas 3 out of 8 were downregulated in 372S. As expected, the phenylpropanoid pathway, which produces secondary metabolites such as flavonoids and lignins, was upregulated in 305R (Additionalfile10: Figure S3). In the sugar pathway, 33 DEGs out of 42 DEGs in 305R were upregulated, whereas only 14 out of 24 were upregulated in 372S. Clearly, sugar pathway in 305R was stimulated by the fungus infection. In a detail, there was a clear upregulation of genes involved in the conversion from glucose to sucrose and fructose in 305R (Fig.4) but not in 372S (Additionalfile11: Figure S4). This corresponds well with increased sugar compounds in metabolite analysis.

### Identification of NBS-ARC genes and their responses to *P. capsici*

A total of 823 candidate NBS-ARC domain proteins were identified by searching the HMM file (PF00931) against the whole-genome peptide sequences. The number increased to 1226 with an E value<0.01 when using the pepper-specific HMM file. Finally, we identified 571 NBS-ARC domain proteins after removing short amino acid sequences. Complete assessment using the CD-Search Tool indicated 390 proteins with a complete NBS-ARC domain. As expected, all the NB-ARC domain genes were clustered among the pepper genomes, particularly at chromosome arms (Additionalfile12: Figure S5). These proteins were grouped according to the repetition and position of NBS-ARC, TIR, CC, LRR, RPW8 as well as coiled coil domain of the potato virus X resistance (RX-CC, abbreviated as Cx in this study) (Table3; Additionalfile6: Table S6; Additionalfile7: Table S7). The conserved domains and motifs as well as the gene structure of all the NBS-ARC genes were analyzed (Additionalfile13: Figure S6). In the pepper genome, there are only 3 TIR-NBS genes, which is notably fewer than in other higher plants. In addition, the three TIR-NBS proteins did not have other representative domains. Among the non-TIR-NBS genes, 204 proteins have LRR domains that may play a role in the recognition of pathogenic effectors. Large variance is noted in the number of LRR domains, which implies coevolution with diseases. For example, one CxNL-type protein (CA01g31440) had as many as 12 LRR domains. CC domains appear frequently in pepper NBS-ARC proteins. There were 118 proteins with CC domains and another 193 proteins that did not have CC domains but had Cx domains. Only 23 proteins had RPW8 domains.

**Table 3:** A summary of NBS-ARC classification in peppers

Group	Num.C ^a^	Num.IC ^b^	Sum
N-type	90	84	174
NL-type	44	36	80
CN-type			
CN	42	15	57
NC	3	0	3
CNC	1	0	1
CxN	83	33	116
CxNCxN	2	0	2
NCxN	1	0	1
CNL-type			
CNL	38	1	39
NLC	0	2	2
NLCL	2	1	3
CxNL	63	3	66
TN-type			
TN	1	1	2
TNT	1	0	1
NT	1	0	1
PN-type			
PNL	2	0	2
PCN	3	2	5
PCNL	8	0	8
PCxN	1	2	3
PCxNL	4	0	4
PCxPN	0	1	1
Total	390	180	570

^a^ num. C, Number of proteins with a complete NBS-ARC domain; ^b^ num. IC, number of proteins with incomplete NBS-ARC domain

**Fig. 5:**
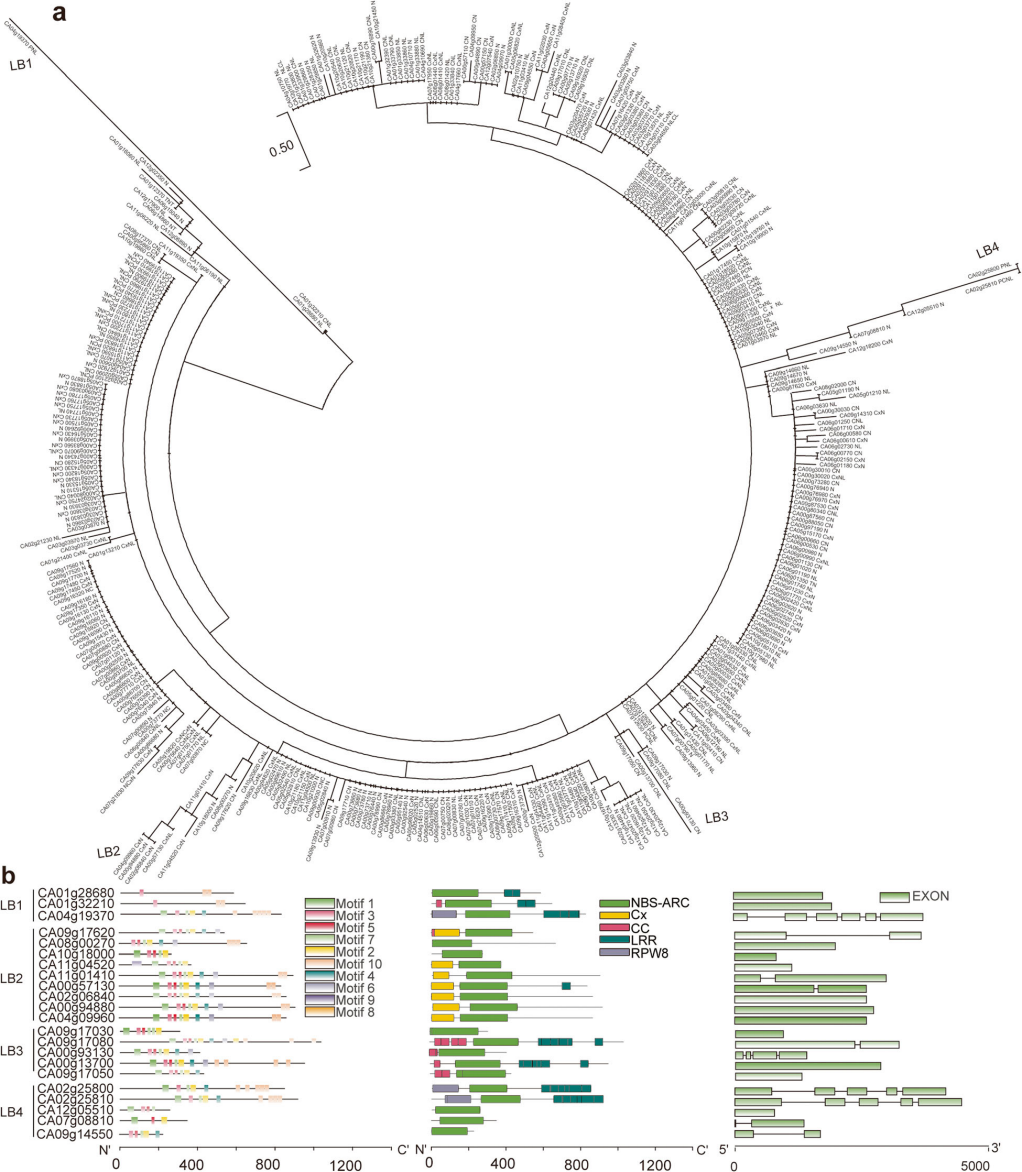
Tree topology of NBS-ARC genes in the molecular phylogenetic analysis. **a** The evolutionary analysis was conducted in MEGA7 using the maximum likelihood method. **b** Conserved motifs and domains as well as the gene structure of genes showing long branches (LB1-LB4) in the tree topology. Conserved domains were determined by CD-Search Tool and HMM tool; motifs were determined by MEME search

**Fig. 6:**
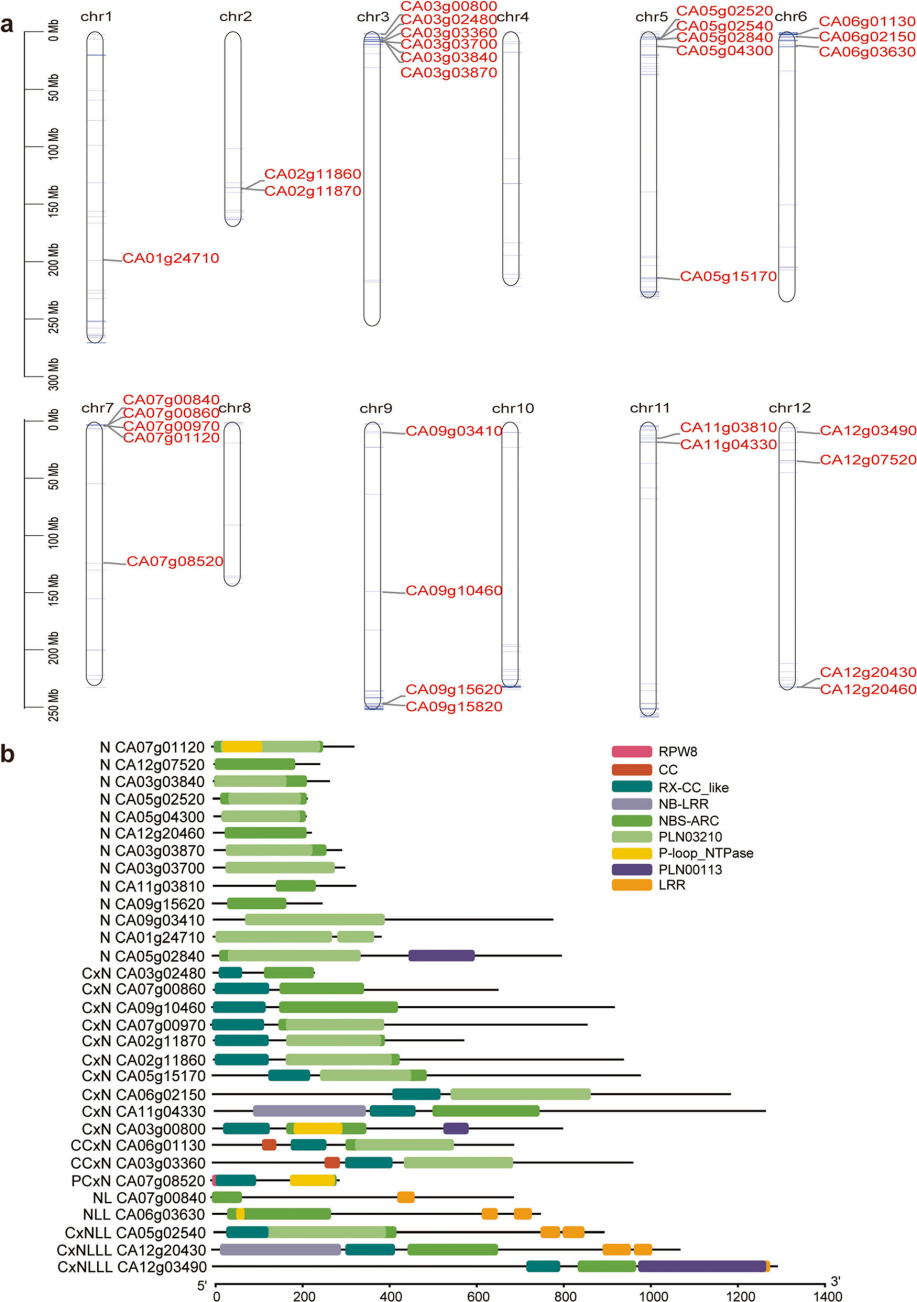
Differentially expressed NBS-ARC genes in *P. capsici* infection. **a** Position of the NBS-ARC DEGs on the chromosomes. **b** Conserved motifs and domains as well as the gene structure of NBS-ARC DEGs. Conserved domains were determined by CD-Search Tool and HMM tool; motifs were determined by MEME search

The polygenetic tree indicates that NBS-ARC domain genes in the same cluster on chromosomes have high identity, e.g., genes on chromosomes 6 and 11 (Fig.5a). Interestingly, genes with long branches, e.g., CA04g19370, CA04g09960, CA00g93130, and CA02g25810, might experience the acquisition of CC, Cx, LRR, or RPW8 domains (Fig.5b). In total, 32 NBS-ARC genes exhibit a significant response to *P. capsici* infection, which were mainly clustered on chromosomes 3, 5, and 7 (Fig.6a). Among them, only 2 had a CC domain, 1 had an RPCW8 domain, and 5 had an LRR domain (Fig.6b). The 5 NB-LRR genes are probably *P. capsici* isolate-specific.

### Response of QTL *Pc5.1* to *P. capsici*

**Fig. 7:**
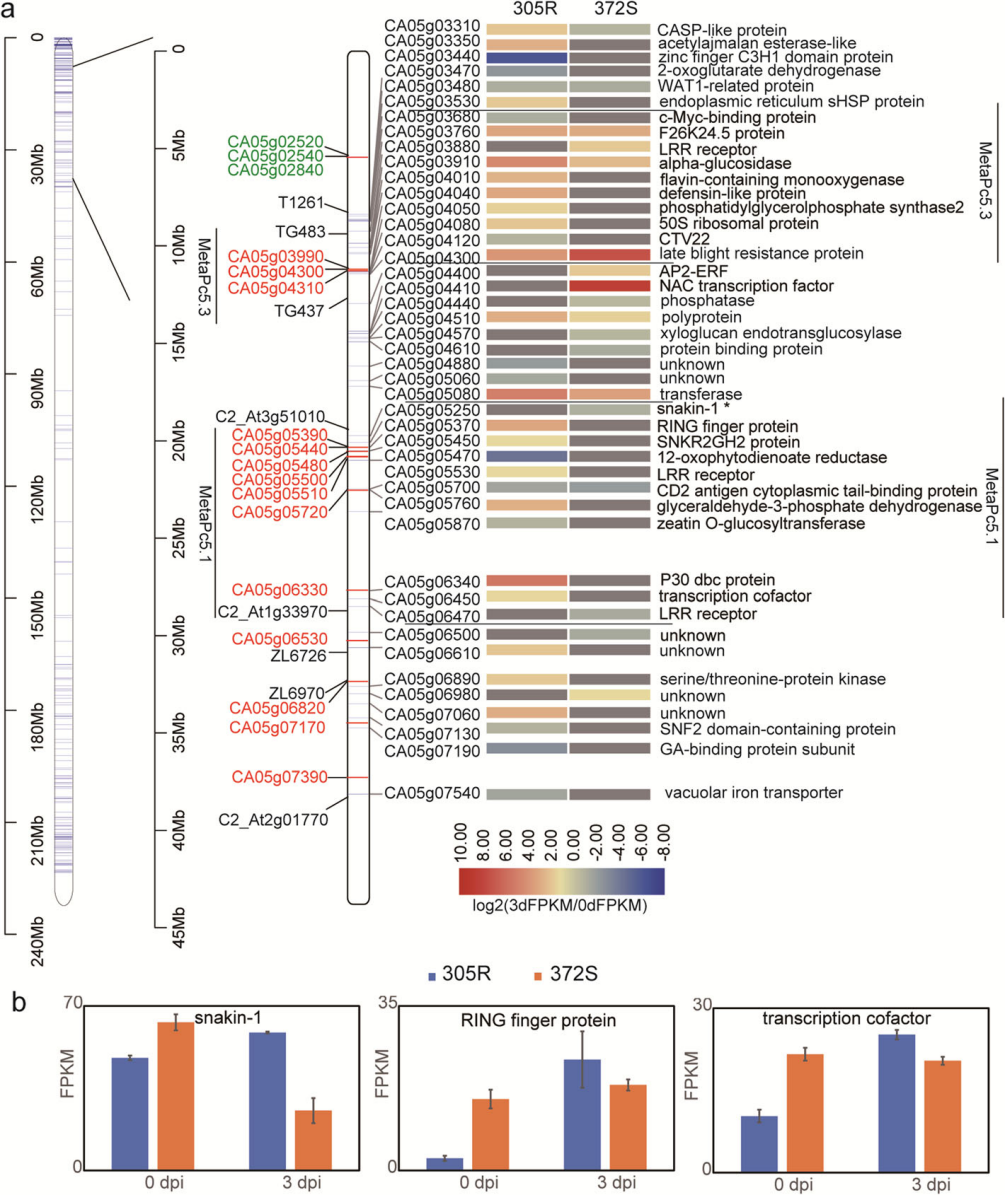
Response profiles of genes positioned within *Ext-Pc5.1* (between T1261 and C2_At2g01770). **a** All the NBS-ARC genes at *Ext-Pc5.1* were labeled in red. All the DEGs at *Ext-Pc5.1* are shown on the right of the chromosome with a transcription heatmap and gene annotation. There were three NBS-ARC DEGs nearing *Ext-Pc5.1*. **b** Three DEGs at Meta Pc5.1 showed different dynamic profile in *P. capsici* infection

QTL *Pc5.1* is known a major and broad spectrum QTL [24]. We converted the genetic positions of molecular markers into physical positions in the CM334 genome by BLAST primer sequences or marker sequences against genome sequences (Fig.7a). We coordinated the *Meta-Pc5.1* locus (between markers C2_At1g33970 and C2_At3g51010) and the adjacent *Meta-Pc5.3* locus (between markers TG483 and TG437). This chromosome segment positioned 8.35Mb - 38.13Mb on chromosome 5 (between markers T1261 and C2_At2g01770) is taken as an extended *Pc5.1* (*Ext-Pc5.1*) in this study.

A total of 44 DEGs were identified at *Ext-Pc5.1* among which 34 DEGs were identified in 305R and 18 in 372S, indicating a stronger response in 305R than in 372S. In a detail, there were 11 DEGs at *Meta-Pc5.1*, 10 DEGs at *Meta-Pc5.3* and 23 DEGs at the surrounding and conjunction regions. At *Ext-Pc5.1*, there were a total of 14 NBS-ARC genes but only one (CA05g04300) of them responded to *P. capsici*, which was induced in both 350R and 372S. Moreover, this *R* gene positioned at *Meta-Pc5.3* and it is N-type. All the candidate genes including *R* genes and other genes at *Pc5.1* proposed in literatures did not show any significant response to *P. capsici* infection (see discussion) [25,27]. And there was no significant difference of these candidate genes in transcription level between 305R and 372S (data not shown). At *Meta-Pc5.1*, we found 3 DEGs showed different dynamic profile in the *P. capsica*-infection including *snakin-1* (*SN1*), RING finger protein, and a transcription cofactor. Basically, their expression level was increased in 305R and decreased in 372S after inoculation(Fig. 7b). *Snakin-1* (*SN1*) is a well-known pathogen defense-related gene, which is characterized by its in vitro activity and targeted pathogens in wide-spectrum [31]. No SNP at coding regions was detected by comparing *SN1* transcripts of 372S and 305R (data not shown).

## Discussion

Transcriptome comparison between resistant material and susceptible material enables us to discriminate the gene response, which should account for the difference in resistance. A recent study of the CM334 root transcriptome proposed that the upregulation of the phenylpropanoid biosynthesis pathway may play a role in resistance (Li, 2020). We also observed the difference in the response of phenylpropanoid biosynthesis pathway between 305R and 372S (Additional file 9: Fig. S3). This differential response is not *P. capsica*-specific and it looks like a common phenomenon in plant resistance against diseases [32, 33]. Indeed, many other clear differences exist between 305R and 372S in addition to the phenylpropanoid pathway. Valine, leucine and isoleucine are branched-chain amino acids (BCAAs) and play roles in plant growth, development, defense, and flavor [34]. The degradation pathway of BCAAs mainly occurs in mitochondria, and its downregulation may help maintain increased BCAA levels and enhance defense. Compared with 372S, which shows a major response in the process of metabolism and oxidation, the dynamic change in the 305R transcriptome is enriched in nucleic processes, such as cell proliferation, DNA replication, nucleosome assembly and transcription regulation, which provides an interesting view about pathogen resistance. We also observed a strong response of epigenetic modification about DNA, histone, and chromatin in 305R but not in 372S. Many reports support the participation of epigenetic mechanisms in plant tolerance against biotic stress [35, 36]. NBS-LRR and pentatricopeptide repeat-containing protein were enriched in processes of DNA methylation and histone methylation, which may generate phased secondary small interfering RNAs (phasiRNAs). The interaction of *R* gene and miRNA-phasiRNA cascade contributes to the delicate control of *R* gene-mediated disease resistance [37]. It is very necessary to profile the dynamic epigenetic change of DNA methylation, histone modification, chromatin remodeling, and small RNA in *P. capsica*-infection in the future.

Notably, sugars including sucrose, tagatose, fructose and mannose were upregulated by pathogen infection in 305R but not in 372S, which was due to the differential response of sugar pathway-related genes in transcriptome. Sugars play roles as energy, carbon sources, and signaling molecules in plant-pathogen interactions [38, 39]. Some sugar pathway genes function as PR proteins, such as cell wall invertases (CWIs), which convert sucrose into glucose and fructose [40]. Many reports show the positive correlation of sugar accumulation with plant resistance against pathogenic microorganisms. For example, pretreatment with sucrose enhances resistance, and accordingly, rice plants overexpressing exotic PR genes accumulate more sucrose in leaves [41]. Apple adopts sorbitol to modulate resistance against fungal disease via NLR genes, and silencing synthesis results in a decrease in resistance [42]. The differential response of sugar accumulation and pathway genes was also observed in soybean infections when comparing resistant and susceptible materials [33]. Here, it is the first-time that sugars were reported to associate with *P. capsica*-resistance. Rapid upregulation of sugar after infection may be involved in lignification of the root cell wall or activate the downstream defense reaction as a part of plant immunity.

We identified a total of 571 NBS-ARC domain genes, 390 of which had a complete NBS-ARC domain. Before our study, only dozens of NBS-ARC genes were identified by degenerate PCR amplification [15, 16]. This is due to either the limitation of degenerate PCR or the absence of genomic sequences at that time. Although the NBS-ARC domain is conserved, sequence variance due to rapid evolution of the gene family is noted; therefore, a large portion of genes cannot be detected by degenerate PCR method. Pepper has an impressively large number of NBS-ARC genes when compared with other reports using similar mining approaches [43]. LRR is the most variable domain of NBS-ARC protein and is critical for recognition specificity in ETI defense reaction [44]. There was a total of 204 NBS-ARC genes possess LRR domains (NB-LRR), however, only 5 of them show significant response. These five NB-LRR genes might be specific to *P. capsici* isolate used in this study. Moreover, there are many more *P. capsici* inducible NBS-ARC genes in 305R than in 372S, which might mean an effective ETI defense reaction in 305R.

Unexpected novel QTLs were occasionally reported, e.g., on chromosome 10, when using a novel isolate of *P. capsici* [26, 45]. Nevertheless, many more studies from the 1990s suggest a wide spectrum QTL on chromosome 5 regardless of resistance resources and QTL mapping strategies [18,23]. Mallard et al. (2013) performed a meta-analysis, compiled QTL mapping work published before, and finally determined three consensus QTLs on chromosome 5 among which *Meta-Pc5.1* (positioned 19.4829.03Mb) and *Meta-Pc5.3* (positioned 9.3013.83Mb) are close to each other on the short arm [24]. Recent studies confirmed again this conclusion. A single dominant gene, *CaPhyto*, was positioned at 30.9832.43Mb (originally 29.1030.18Mb in the Zunla-1 genome) [25]. With the combination of biparental QTL mapping and GWAS, the wide spectrum QTL *Pc5.1* (originally *QTL5.2*) against 3 *P. capsici* isolates was positioned at 27.3Mb [27]. Another two isolate-specific QTLs were identified near a region of 18.719.5Mb (*Pc5.1* originally *QT5.1*) and 34.637Mb. Most recently, *Pc.5.1* at 27.16Mb of chromosome 5 was detected again in the resistance to 4 isolates [26]. Taking into consideration these above QTL positions, we dissected the response of *Ext-Pc5.1* with a region 8.3538.13Mb.

Despite the effort in genetic analysis, very little is known about the detailed genetic mechanism. In the resistant resource PI201234, two genes, CA05g06820 (RPP13-like, Capana05g000769 in the Zunla-1 genome) and CA05g06770 (serine/threonine-protein kinase BRI1-like2, Capana05g000764 in the Zunla-1 genome), of *Pc5.1* were proposed to control resistance [25]. At *Pc5.1* of CM334, 2 RPP13-like genes, 3 RLK genes, and a systemic acquired resistance (SAR)-related gene are suggested candidate genes participating in resistance regulation [27]. Chunthawodtiporn et al. (2019) again proposed the late blight resistance genes CA05g05390, CA05g05440, and CA05g05720 [26]. All of these studies, together with the QTL on chromosome 10, led to a consensus about the role of the *R* genes in *P. capsici* resistance. Nevertheless, we did not detect any differential expression of all the candidate genes proposed at *Pc5.1*. Three other *R* genes (CA05g02520, CA05g02540, CA05g02840) at positions 5.476.74Mb and a cluster of LRR receptor-like genes (CA05G02270, CA05G02310, CA05G02340, CA05G02350) located at 4.54.7Mb showed significant response. However, they are too far from the consensus Meta-*Pc5.1* and other published *Pc5.1*. Instead, they are likely located at a rare locus *CC_Pc5.4* [23]. These differential expressions are probably *P. capsici*-specific because their expression was not affected by inoculation with *Phytophthora infestans*, pepper mottle virus, or tobacco mosaic virus [46]. Reverse genetic studies stress the function of many other factors in *P. capsici* resistance, e.g., CaRGA2 [47], EDS1 [11], CaChiIV1 [48], CaAP2/ERF064 [49], and squamosal promoter binding protein (SBP) [50]. However, no significant response of these genes was identified at the locus *Pc5.1*.

*Snakin-1* (*SN1*) gene (CA05g05250) encoding a cysteine-rich antimicrobial peptide (AMP) might contribute to the broad-spectrum QTL. Snakin protein which was also called gibberellin stimulated peptide is firstly and thoroughly studied in potato since 1990s [51]. Many studies have proved that snakin proteins show strong antimicrobial activity in vivo and in vitro. Over expression of potato SN1 can increase resistance against fungal pathogens in wheat [52], alfalfa [53], and lettuce [54]. In pepper, CaSnakin protein was induced by root-knot nematode infection and play a role in resistance to nematode [55]. More genetic evidences need to be provided in the future to clarify it function in *P. capsici*-resistance.

## Conclusions

We screened out 10 pepper materials showing high resistance to *P. capsica.* Primary metabolites of infected root were detected by GC-MS and sugars were greatly upregulated by *P. capsica* infection in 305R but not in 372S.We dissect the response of *Ext-Pc5.1* and *R* genes in *P. capsici* infection in transcriptome analysis. A total of 570 NBS-ARC genes were identified in pepper CM334 genome, among which 34 genes were affected by the infection, but only 5 genes belonged to NB-LRR type. These NB-LRR DEGs were not positioned at *Ext-Pc5.1* and all the candidate genes proposed in previous publications did not show any response in *P. capsici* infection. These results provide new insights about the role of *R* genes at the broad-spectrum *Pc5.1* QTL. At least, we should not ascribe the *P. capsici*-resistance controlled by *Pc5.1* to the transcriptional response of *R* genes. We propose *SN1* at *Meta-Pc5.1* as the candidate gene controlling the wide-spectrum resistance. In addition, we found interesting and very clear response of sugar metabolism and processes of epigenetic modification systems, which provide a new insight into the resistance mechanism.

## Materials and methods

### Plant materials

Phytophthora-resistant peppers were screened from 160 germplasm materials. All pepper seedlings were cultured in plug trays with 50 cells in a culture room. Ten seedlings for each accession were surveyed in the resistance investigation at the stage of 6 leaves. The known pepper CM334 with high resistance was used as a positive control. Two resistant accessions, 304R and 305R, and two susceptible accessions, 370S and 372S, were identified and used in metabolite analysis and RNA-seq. Entire roots from each seedling were collected at 0 dpi, 3 dpi, and 5 dpi according to the development of the dynamic symptom. There was no symptom at 0 dpi; symptom appeared at 3 dpi; the whole plant became wilting at 5 dpi. Roots from 5 seedlings were mixed and homogenized as one sample. Triplicate samples were obtained at each sampling time point. All the samples were used to analyze the dynamic changes in root metabolites. The same samples at 0 dpi and 3 dpi were also used in RNA-seq.

### *P. capsici* infection and resistance identification

The *P. capsici* isolate named LJYM1 was provided by Sichuan Academy of Agricultural Sciences, which were collected from Chengdu, China and was identified as physiological race 2 [56]. The inoculation method of disease PPR followed that described by Kimble [2]. Subcultures were performed on potato dextrose agar (PDA) medium at 27C in the dark. Proliferation culture was performed on V8 medium in the dark until the plates were uniformly covered with mycelia. Zoospores were then induced in light and collected to make zoospore solutions with a concentration of 10^5^ spores/ml. Five milliliters of zoospore solution was injected into the soil around the basal stem of pepper seedlings at the 6-leaf stage. Injury of disease PPR was assessed from 0 to 10 dpi following the standard method described by Li et al. (2006) [57]. Resistance assessment was performed according to the index of disease injury at 5 dpi. High resistance (HR) was determined by very low disease index (010); Resistance (R) was determined by low disease index (1025); moderate resistance (MR) was determined by moderate disease index (2550); nonresistance (NR) was determined by high disease index (50100).

### Metabolite extraction and GC-MS analysis

The method of metabolite extraction and detection follows Desbrosses et al. (2005) with modification [58]. In brief, each root sample was dried by a vacuum freeze dryer at 40C, and 100mg tissues were pulverized in a 2-ml tube using a slender glass pestle. Ground samples were suspended in 540l precooled methanol plus 60l ribitol in methanol (2mg/ml), which was used as an internal standard. The tube was vortexed and then put in a water bath at 4C for 30min with ultrasonic treatment. After adding 600l of water and 300l of chloroform, the samples were vortexed and then centrifuged at 14,000rpm for 10min at room temperature. We then transferred 400l aliquots of the aqueous phase to 2ml tubes and dried them at room temperature by vacuum centrifugation. The precipitate metabolites were suspended and derivatized by methoxyamination in 40l methoxyamine hydrochloride in pyridine (20mg/ml) for 2h at 37C. Subsequent trimethylsilylation was performed at 37C for 30min after adding 70l MSTFA solution. The derivatized solution was filtered through a 45-m membrane after adding 900l hexane. All chemicals and reagents were of analytical grade.

Qualitative detection of primary metabolites in roots was performed using an Agilent GC-MS (7890A-5975C) equipped with an HP-5MS column (30.0m0.25mm0.25m). One l sample was injected in split-less mode. The oven temperature was initially maintained at 100C for 1min, increased to 184C at a rate of 3C/min, increased to 190C at a rate of 0.5C/min, and finally increased to 280C at a rate of 15C/min. Mass spectra were collected at a scanning range of 40600m/z. Each metabolite was identified by retention indices and comparison of mass spectra with a reference mass spectral library (NIST2011, Gaithersburg, MD, USA).

### RNA-seq and differential gene expression caused by *P. capsici* infection

Root samples were ground into power in liquid nitrogen, and 100mg of each sample was used to extract total RNA using the TRIzol method as previously described [59]. One microgram of total RNA was purified via DNase I digestion. Poly(A) mRNAs were enriched using oligo (dT) magnetic particles. The obtained mRNA was fragmented via metal hydrolysis in 1 fragmentation buffer (Life Technologies) and then ligated to an RNA oligonucleotide adaptor. The ligation products were used to generate first-strand cDNA via reverse transcription (RT)-PCR. Then, short PCR was performed to amplify the cDNA to obtain sufficient quantities of DNA products. A single A nucleotide was added to the 3 ends of cDNAs to facilitate ligation with adapters. Sequencing was performed using an Illumina Genome Analyzer IIx according to the manufacturers instructions.

Reads with adapters, reads with greater than 10% unknown bases, and low-quality reads were filtered to obtain clean reads. Clean reads were mapped to the CM334 genome (ftp://ftp.solgenomics.net/genomes/Capsicum_annuum/) using HISAT2 [60] and assembled the transcripts with mapped reads using StringTie [61]. For the analysis of protein-coding genes, only uniquely mapped reads were used, and the transcript levels were calculated as fragments per kilobase of transcript per million fragments (FPKM) using the Cufflinks software package [62]. DEGs were identified using DEseq [63], and the criteria were FDR<0.01 and fold-change2.

### Identification of NBS-ARC domain genes

The genomic assembly and annotation of CM334 were downloaded from the Sol Genomics Network (ftp://ftp.solgenomics.net/genomes/Capsicum_annuum/). NBS-ARC candidate proteins were screened using HMMER v3 as described by Lozano et al. (2015) with minor alterations [43]. A hidden Markov model (HMM) of the NBS-ARC family (PF00931) from the Pfam database (http://pfam.sanger.ac.uk/) was used to search NBS-ARC domain proteins from genomic amino acid sequences using the function hmmsearch of HMMER v3. A pepper-specific NBS-ARC HMM was created from the obtained high-quality protein set (E-value<110^55^) using the function hmmbuild of HMMER v3. The obtained specific HMM file was used to search NBS-ARC domain proteins again from genomic amino acid sequences using the function hmmsearch. Candidate proteins with an E-value<0.01 and lengths shorter than 200bp were discarded. The HMMs of NBC-ARC (PF00931), TIR (PF01582), RPW8 (PF05659), and LRR (PF00560, PF07723, PF07725, and PF12799) from the Pfam database were merged into one HMM by the function hmmpress and used to mine the corresponding domains by the hmmscan HMMER v3 suite. Paircoliled 2 (http://cb.csail.mit.edu/cb/paircoil2/paircoil2.html) was used to identify the CC domain with a P-score cutoff of 0.03 and minimum search window length of 21. Conserved domains were confirmed by the Batch Web CD-Search Tool (https://www.ncbi.nlm.nih.gov/Structure/bwrpsb/bwrpsb.cgi) and Multiple Expectation for Motif Elicitation (MEME). All the identified NBS-ARC domain proteins were grouped according to the number and position of NBS-ARC, CC, TIR, RPW8, LRR, and RX-CC_like. Tree topology of NBS-ARC genes was constructed by MEGA7 [64]. The visualization of gene structure and domain pattern was done by using TBTools [65].

## Supplementary Information

**Additional file 1: Table S1.** Assessment of resistance against *P. capsici* in peppers.

**Additional file 2:Table S2.** Raw data of RNA-sequencing.

**Additional file 3:Table S3.** DEGs identified in 305R root transcriptome after *P. capsici* inoculation.

**Additional file 4: Table S4.** DEGs identified in 372S root transcriptome after *P. capsici* inoculation.

**Additional file 5: Table S5.** DEGs that were enriched in Epigenetic-related biological processes in GO enrichment analysis.

**Additional file 6: Table S6.** Detailed information of each NBS-ARC protein on the representative conserved domains.

**Additional file 7:Table S7.** Classification of NBS-ARC domain proteins in peppers.

**Additional file 8: Figure S1.** Top 20 enrichments in KEEG enrichment analysis.

**Additional file 9: Figure S2.** GO enrichment analysis of DEGs.

**Additional file 10:Figure S3.** Transcription heatmap of genesof phenylpropanoid pathway in KEGG analysis.

**Additional file 11: Figure S4.** The dynamic change of sugar pathway genes after *P. capsici* infection in 372S.

**Additional file 12: Figure S5.** Position of NBS-ARC domain genes on pepper chromosomes.

**Additional file 13.** Protein structure and gene structure of NBS-ARC genes with complete NBS-ARC domains.

## Data Availability

The clean data of RNA-seq were deposited in the NCBI GeneBank under accession number SAMN17915256, SAMN17915257, SAMN17915258, SAMN17915259.

## Abbreviations

DPI: Days post inoculation
DEG: Differentially expressed gene
KEGG: Kyoto encyclopedia of genes and genomes
GO: Gene ontology
PRR: Phytophthora capsici root rot
QTL: Quantitative Trait Locus
PTI: Pattern-triggered immunity
ETI: Effector-triggered immunity
NBS: Nucleotide binding site
ARC: Apoptosis, R proteins, CED-4
LRR: Leucine rich repeat
TIR: Toll/interleukin-1 receptor
RPW8: Resistance to powdery mildew 8
CC: Coiled-coil
RX-CC: Coiled coil domain of the potato virus X resistance
Cx: RX-CC
HR: High resistance
MR: Moderate resistance
NR: Nonresistance
RPP13: Resistance to *Peronospora parasitica* protein 13
SN1: Snakin-1
PR: Pathogenesis-related
