# Supplementary figures and images for: The dissection of R genes and locus Pc5.1 in Phytophthora capsici infection provides a novel view of disease resistance in peppers
Source: BMC Genomics. 2021 May 21;22:372. doi: 10.1186/s12864-021-07705-z (PMC8139160; doi:10.1186/s12864-021-07705-z)

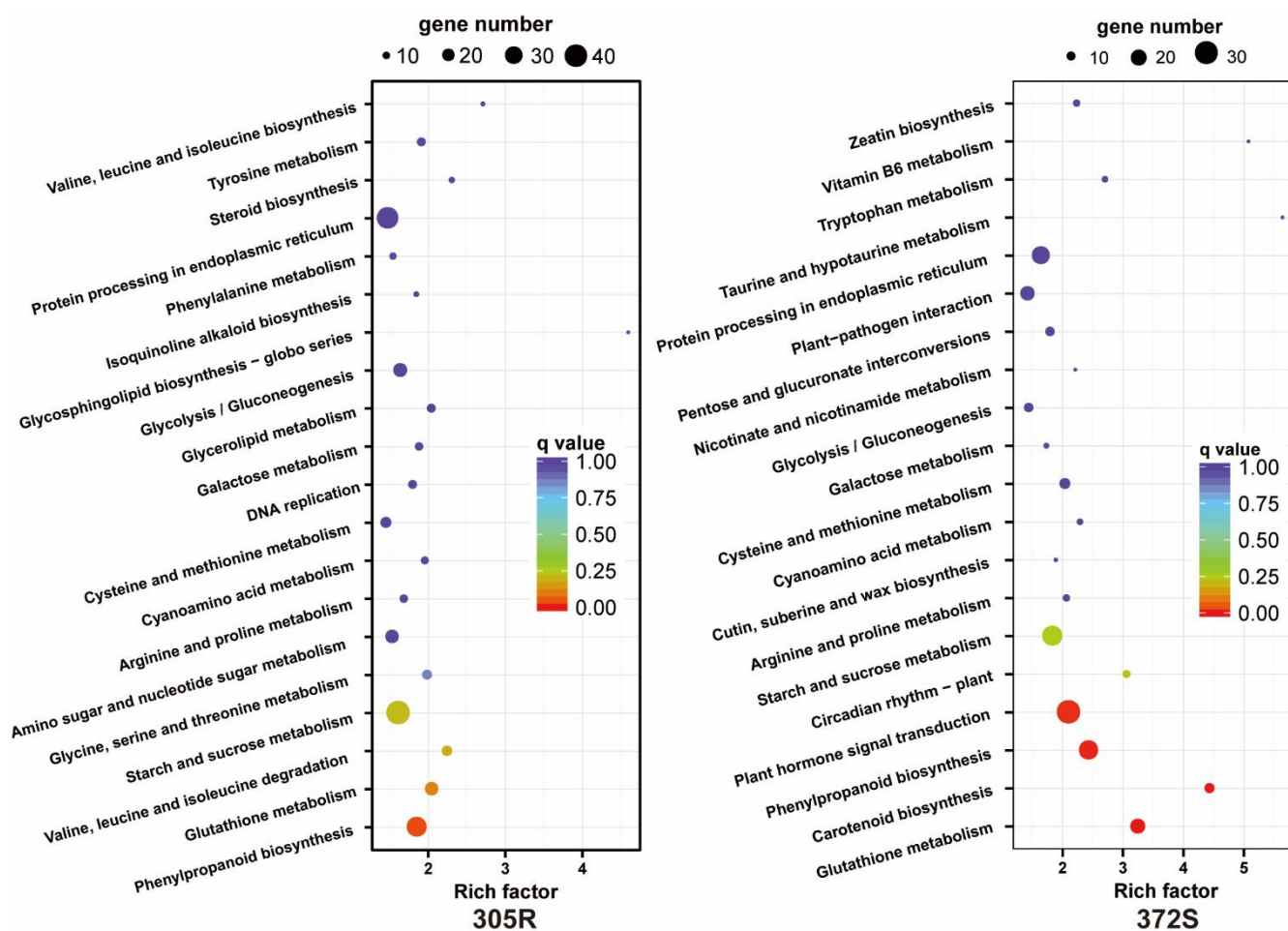

**Figure S1** Top 20 enrichments in KEEG enrichment analysis. Left, 305R; Right, 372S.

Supplement: Supplementary file 8 — Additional file 8: Figure S1. Top 20 enrichments in KEEG enrichment analysis. [file 12864_2021_7705_MOESM8_ESM.pdf]

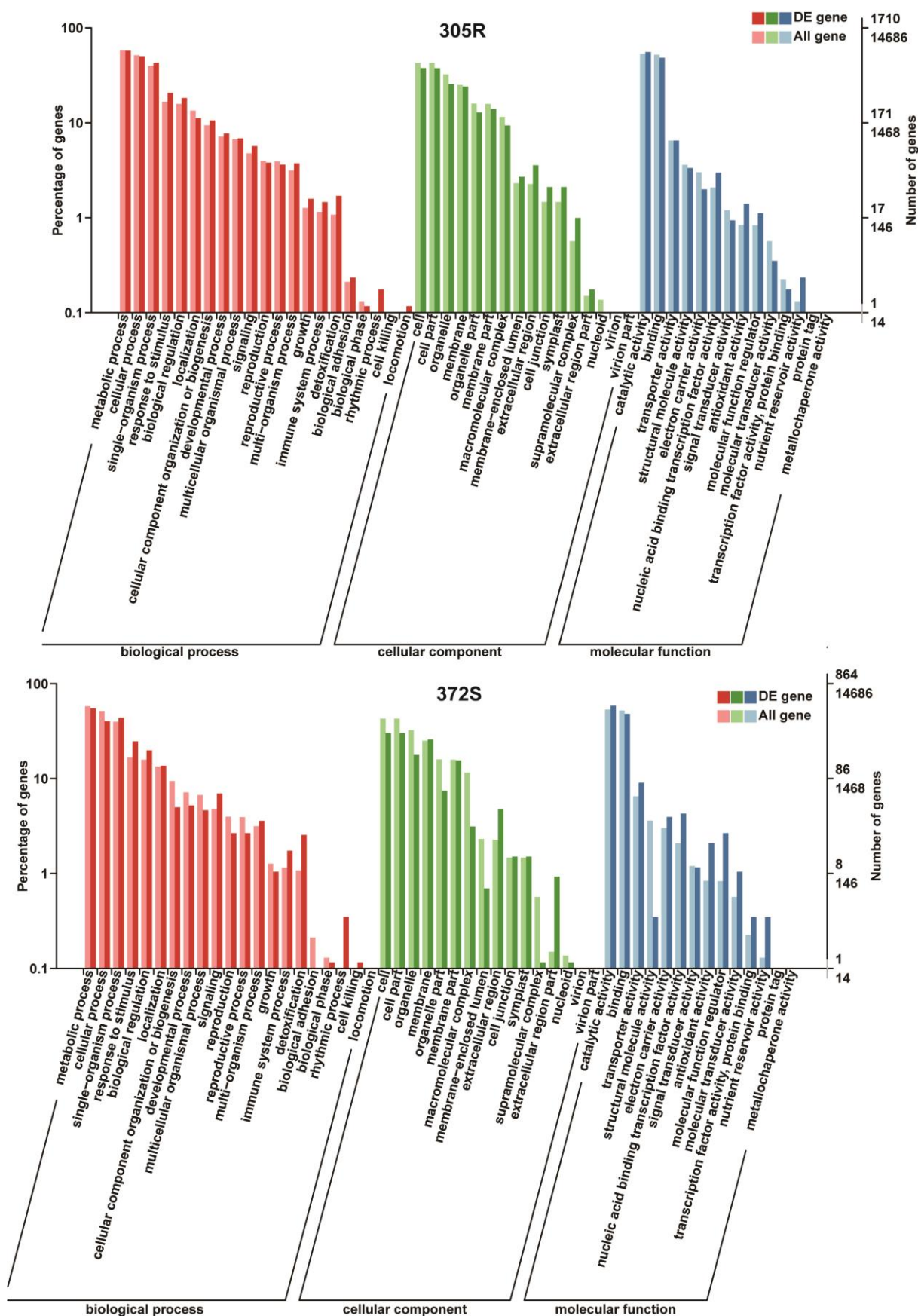

**Figure S2** GO enrichment analysis of DEGs.

Supplement: Supplementary file 9 — Additional file 9: Figure S2. GO enrichment analysis of DEGs. [file 12864_2021_7705_MOESM9_ESM.pdf]

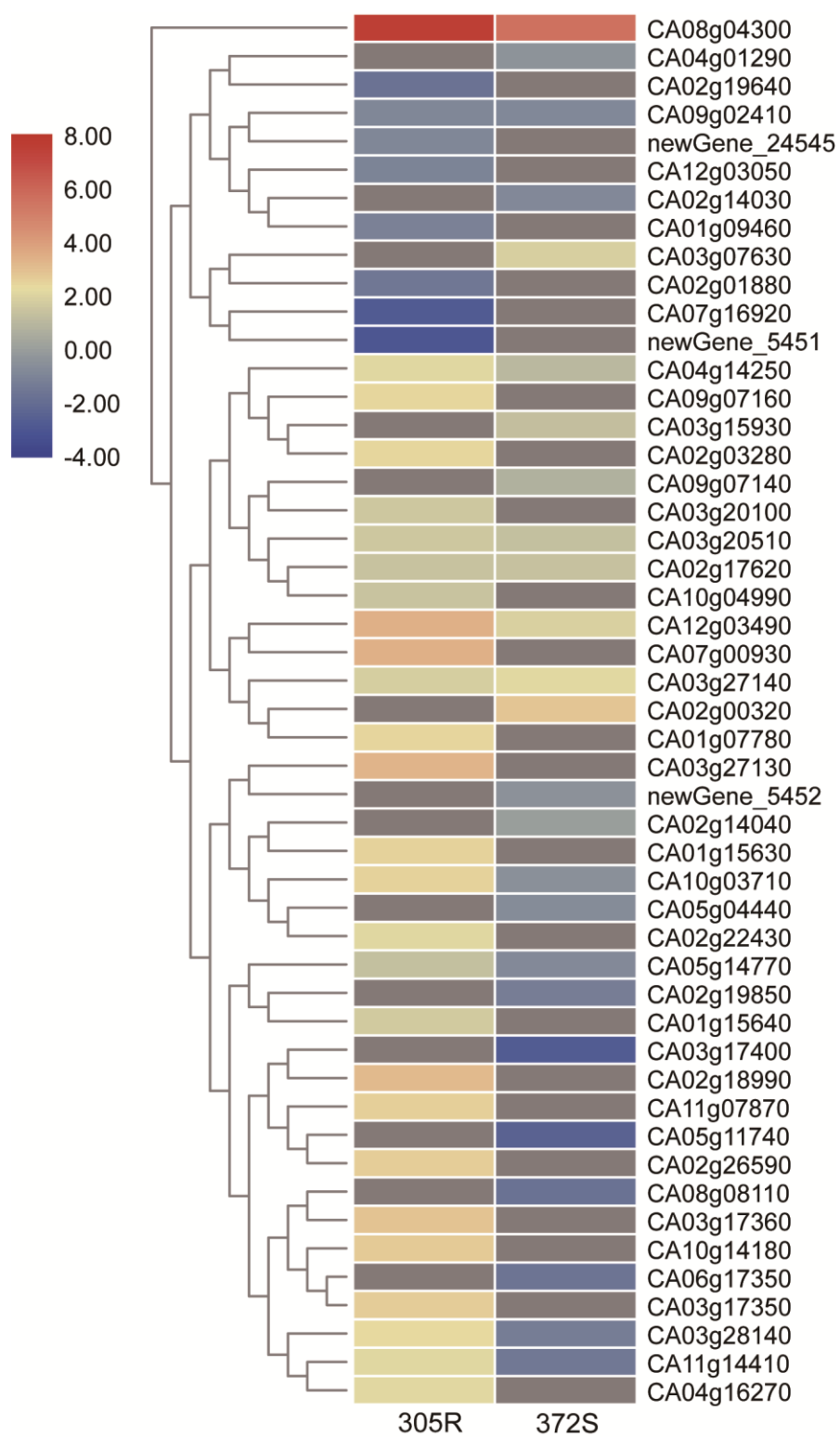

**Figure S3** Transcription heatmap of genes of the phenylpropanoid pathway in KEGG analysis.

Supplement: Supplementary file 10 — Additional file 10:Figure S3. Transcription heatmap of genesof phenylpropanoid pathway in KEGG analysis. [file 12864_2021_7705_MOESM10_ESM.pdf]

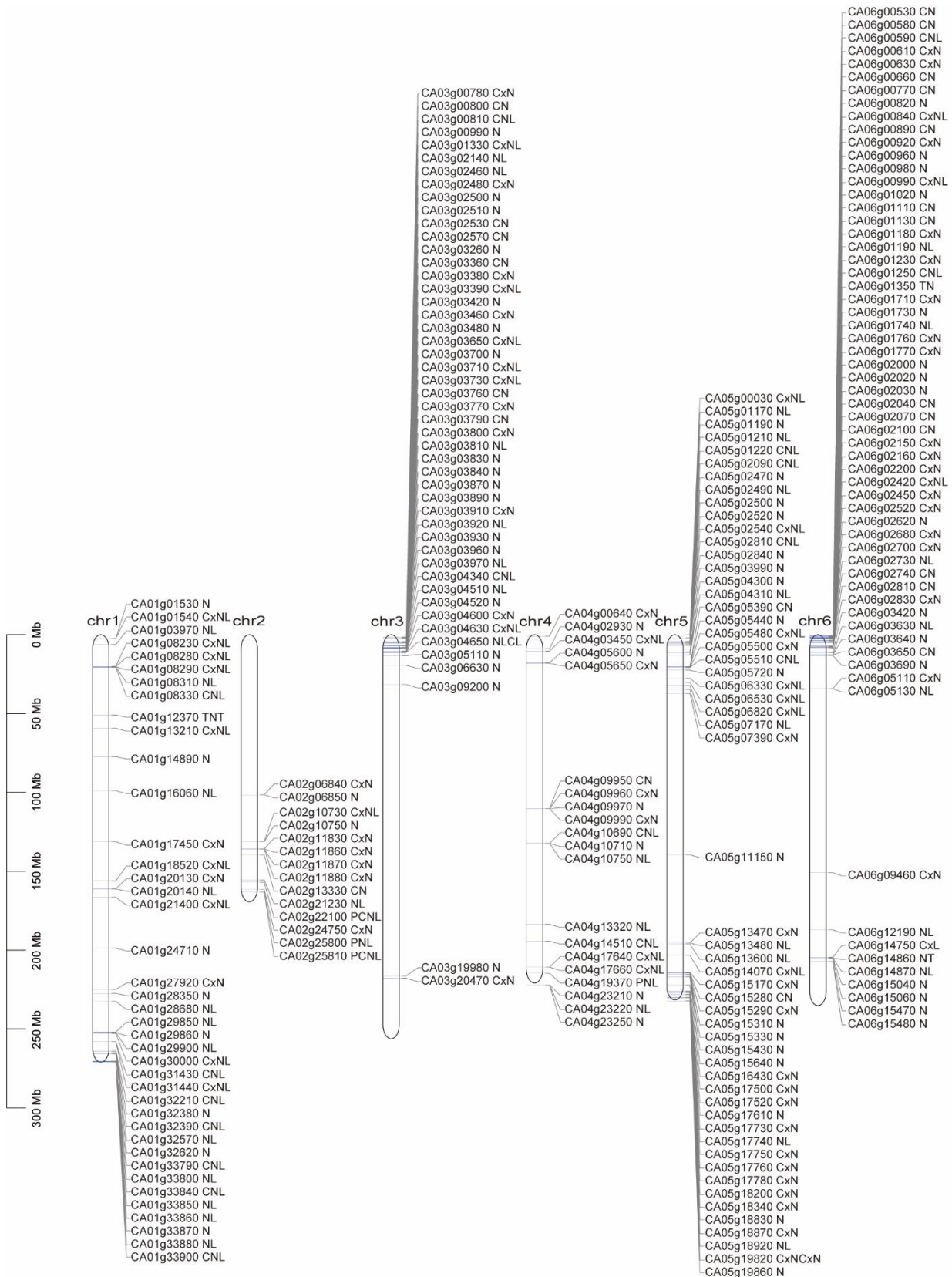

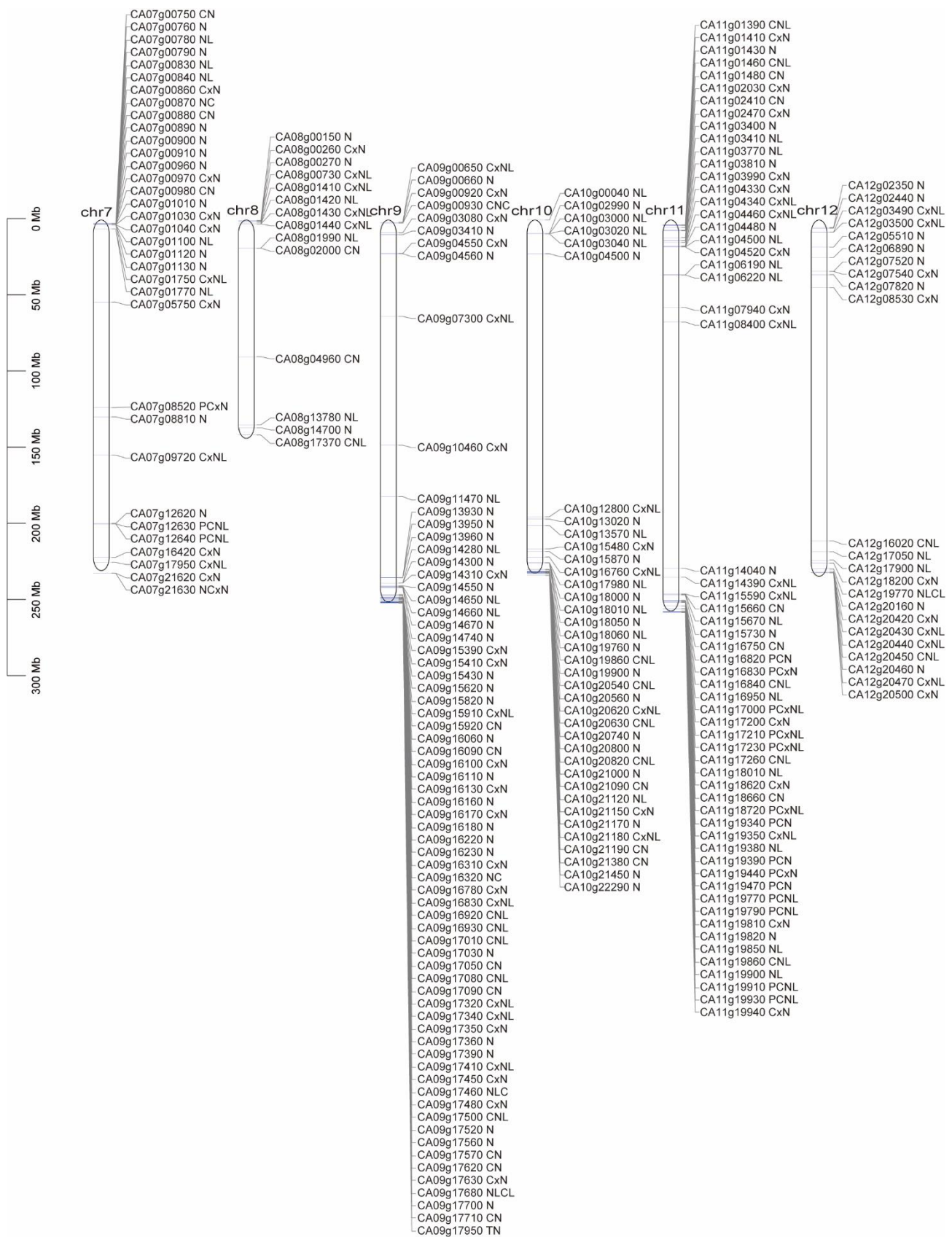

**Figure S5** Position of NBS-ARC domain genes on pepper chromosomes.

Supplement: Supplementary file 12 — Additional file 12: Figure S5. Position of NBS-ARC domain genes on pepper chromosomes. [file 12864_2021_7705_MOESM12_ESM.pdf]
