# Supplementary material for: The dissection of R genes and locus Pc5.1 in Phytophthora capsici infection provides a novel view of disease resistance in peppers
Source: BMC Genomics. 2021 May 21;22:372. doi: 10.1186/s12864-021-07705-z (PMC8139160; doi:10.1186/s12864-021-07705-z)
Supplement: Supplementary file 11 — Additional file 11: Figure S4. The dynamic change of sugar pathway genes after P. capsici infection in 372S. [file 12864_2021_7705_MOESM11_ESM.pdf]

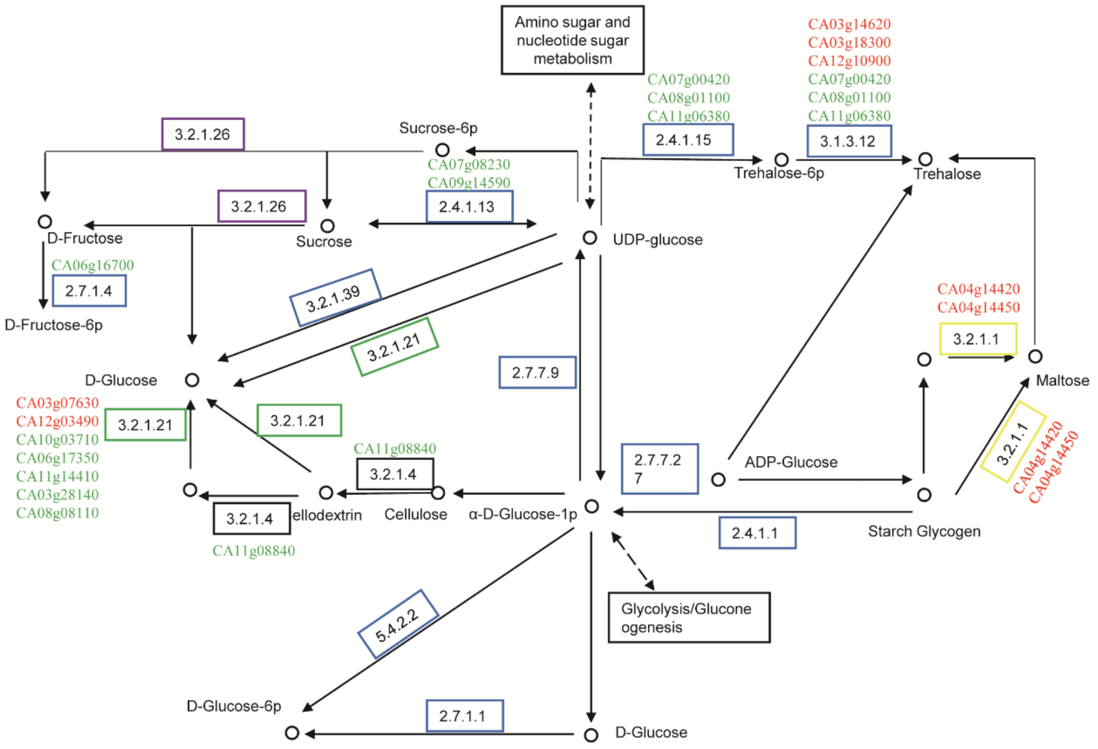

Figure S4 The dynamic change of sugar pathway genes after *P. capsici* infection in 372S. The pathway map was made based on map00500 (Starch and sucrose metabolism) from KEGG PATHWAY Database. Upregulated genes were labeled in red color while downregulated genes were labeled in green color.
