# Supplementary material for: The dissection of R genes and locus Pc5.1 in Phytophthora capsici infection provides a novel view of disease resistance in peppers
Source: BMC Genomics. 2021 May 21;22:372. doi: 10.1186/s12864-021-07705-z (PMC8139160; doi:10.1186/s12864-021-07705-z)
Supplement: Supplementary file 13 — Additional file 13. Protein structure and gene structure of NBS-ARC genes with complete NBS-ARC domains. [file 12864_2021_7705_MOESM13_ESM.pdf]

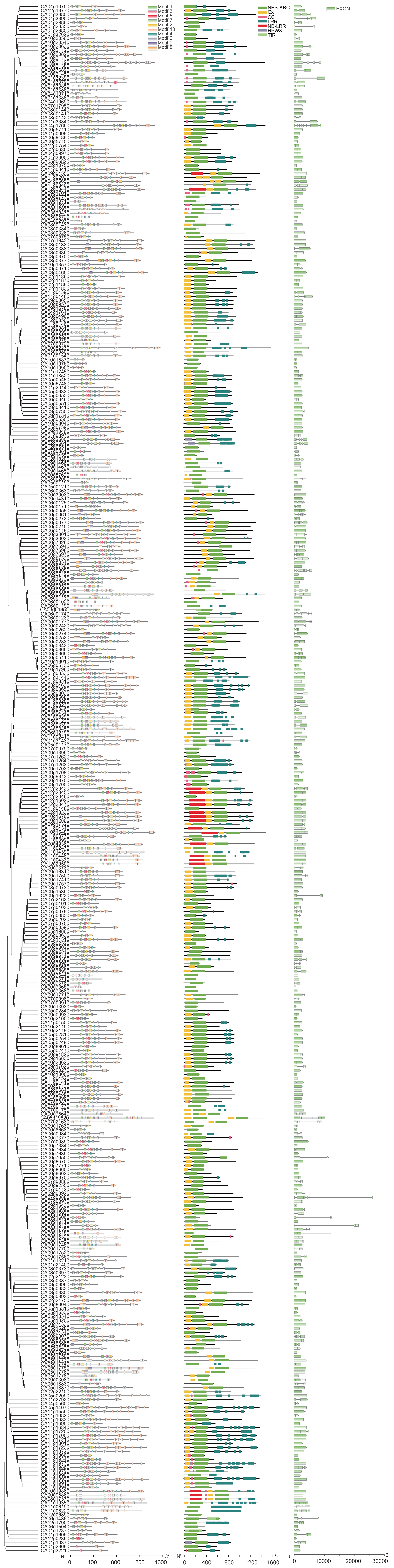

Figure S6 Protein structure and gene structure of NBS-ARC genes with complete NBS-ARC domains. Conserved domains were determined by "CD-Search Tool" and HMM tool; motifs were determined by MEME search.
